# Detection of leukemia gene fusions by targeted RNA-sequencing in routine diagnostics

**DOI:** 10.1186/s12920-020-00739-4

**Published:** 2020-07-29

**Authors:** Marie Engvall, Nicola Cahill, Britt-Inger Jonsson, Martin Höglund, Helene Hallböök, Lucia Cavelier

**Affiliations:** 1 Department of Immunology, Genetics, and Pathology, Uppsala University, Uppsala, Sweden; 2 Clinical genetics, Uppsala University Hospital, SE-751 85 Uppsala, Sweden; 3 Department of Medical Sciences, Uppsala University, Uppsala, Sweden

**Keywords:** Leukemia, Gene fusion, NGS, Targeted RNA sequencing, KMT2A

## Abstract

**Background:**

We have evaluated an NGS-based method to detect recurrent gene fusions of diagnostic and prognostic importance in hematological malignancies. Our goal was to achieve a highly specific assay with a simple workflow, short turnaround time and low cost.

**Method:**

The assay uses a commercially available anchored multiplex PCR panel for target enrichment and library preparation, followed by sequencing using a MiSeq instrument. The panel includes all recurrent gene fusions in AML and ALL and is designed to detect gene-specific fusions without prior knowledge of the partner sequence or specific break points. Diagnostic RNA samples from 27 cases with hematological malignancies encompassing 23 different transcript variants were analyzed. In addition, 12 cases from a validation cohort were assessed.

**Result:**

All known fusion transcripts were identified with a high degree of confidence, with a large number of reads covering the breakpoints. Importantly, we could identify gene fusions where conventional methods had failed due to cryptic rearrangements or rare fusion partners. The newly-identified fusion partners were verified by RT-PCR and transcript-specific qPCR was designed for patient-specific follow-up. In addition, 12 cases were correctly assessed in a blind test, without prior knowledge of molecular cytogenetics or diagnosis.

**Conclusion:**

In summary, our results demonstrate that targeted RNA sequencing using anchored multiplex PCR can be implemented in a clinical laboratory for the detection of recurrent and rare gene fusions in hematological diagnostic samples.

## Background

Chromosomal rearrangements such as translocations, inversions or deletions, can cause breakpoints within genes leading to gene fusions which code for fusion proteins with altered functionality. Gene fusions are frequently seen in leukemia and several of the recurrent gene fusions are required for subgrouping of leukemia and prognostication, according to the WHO classification [1]. One example is the *BCR*-*ABL1* fusion in chronic myeloid leukemia (CML), occurring most commonly as a result of a translocation between the long arms of chromosomes 9 and 22 which gives rise to the “Philadelphia chromosome” [2]. The *BCR-ABL1* fusion produces a fusion protein with increased tyrosine kinase activity. The fusion protein has successfully been targeted with specific tyrosine kinase inhibitors, greatly improving the prognosis of CML patients [3]. Another gene fusion that is effectively treatable is the *PML*-*RARA* fusion in acute myeloid leukemia (AML). This gene fusion expresses a fusion protein which acts as a transcriptional regulator and interacts with ATRA. By increasing the physiological concentration of ATRA through ATRA treatment the PML-RARA fusion protein is degraded [4].

Clinical diagnostic laboratories routinely use an array of methods to detect gene fusions, including chromosome analysis, fluorescence in situ hybridization (FISH), reverse transcriptase (RT)-PCR and Southern blot. Chromosomal rearrangements can have different breakpoints generating various fusion transcripts. Some genes also present multiple fusion partners, e.g. the *KMT2A*-gene (previously known as *MLL*) located at band q23 on chromosome 11. *KMT2A* is commonly rearranged in both pediatric and adult acute lymphoblastic leukemia (ALL) and AML. One hundred thirty-five different fusion partner genes have been described so far, of which *AFF1*, *MLLT1*, *MLLT3*, *MLLT10*, *MLLT4* and *ELL* are the most common [5, 6]. Furthermore, different types of structural rearrangements can be the underlying cause of the *KMT2A* fusions, including translocations, insertions, inversions and deletions.

**Fig. 1:**
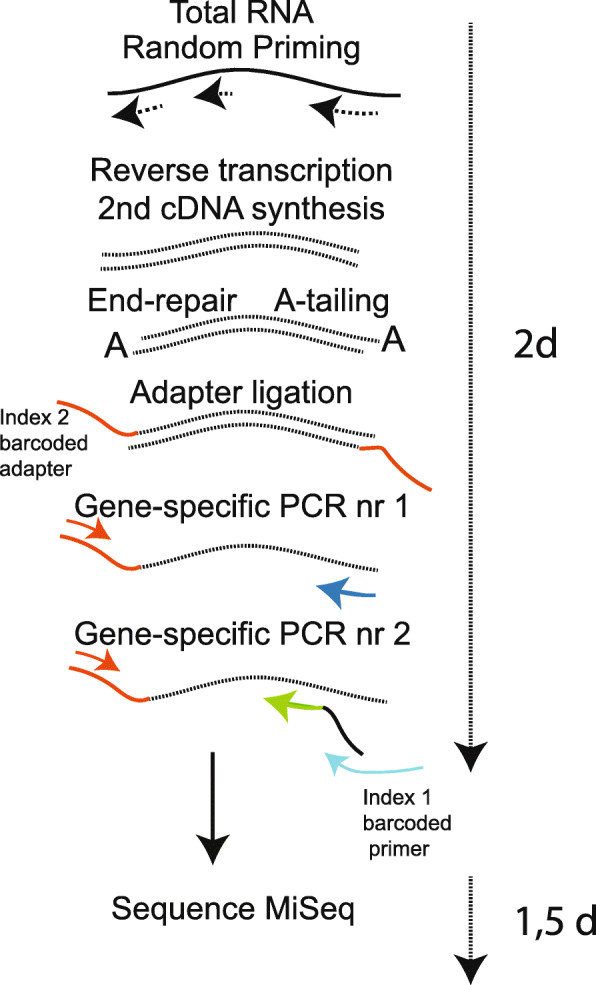
Workflow of targeted RNA sequencing using anchored multiplex PCR

To overcome the labor-intensive methods routinely used to detect gene fusions, especially for the *KMT2A*-gene, NGS-based methods can be applied to screen for gene fusions in patient samples, by sequencing the breakpoints of the fusion. In several studies, mRNA-sequencing has been successfully adopted to detect gene fusions in leukemia, e.g. gene fusions in AML [7] or *KMT2A* fusions in infant ALL [8]. To date, many of these studies have largely focused on using RNA sequencing to detect recurrent gene fusions in large batches of samples collected over time that were subsequently sequenced concurrently in a high throughput fashion. In contrast, clinical genetic diagnostics of leukemia not only requires a low cost per sequencing run but critically demands shorter turnaround time. The requirement of a short turnaround time precludes batching of samples as commonly performed in a research environment. In our laboratory, the turnaround time for FISH screening of recurrent gene fusions in acute leukemia is (at maximum) 5 days. To achieve a comparable turnaround time for gene fusion detection with a relatively low cost per test, we have investigated the use of an NGS-based fusion gene detection assay using a benchtop instrument, the MiSeq from Illumina. To reach the sequencing depth required for sensitive detection of gene fusions, we performed targeted sequencing by enriching for a panel of recurrent gene fusions in leukemia. Anchored multiplex PCR is a method that can be used to enrich cDNA libraries for specific genes (Fig. 1). The method combines gene-specific primers with adapters containing a universal primer binding site to amplify sequences of interest without prior knowledge of the partner sequence or specific break points. For increased amplicon specificity, a nested gene-specific primer is used for a second PCR. The hematological panel comprises 20 genes (Table 1) and covers the recurrent gene fusions in AML and ALL. The library preparation requires a short hands-on-time and the sequence analysis software to detect such gene fusions is freely available. In total, including sample and library preparation, sequencing and data analysis takes less than 5 days. The cost per sample is around 500–600 euro.

**Table 1:** List of genes included in the Archer™ FusionPlex™ Heme Panel version 1 and examples of rearrangements that can be detected

***Gene***	***Examples of rearrangements***	***Included in the study?***	***Number of cases*** ***Primary cohort / validation cohort***
*ABL1 and BCR*	*t(9;22) (BCR-ABL1), other ABL1-rearrangements*	*Yes*	*2 / 1*
*ALK*	*ALK-rearrangements*	*No*	*–*
*CBFB*	*t/inv*(16)*, del*(16) *(CBFB-MYH11), other CBFB-rearrangements*	*Yes*	*1 / 1*
*FGFR1*	*8p11, FGFR1-rearrangements*	*No*	*–*
*JAK2 (5′ and 3′)*	*t(9;12) (ETV6-JAK2), other JAK2-rearrangements*	*No*	*–*
*KMT2A (MLL) (5′ and 3′)*	*All KMT2A-rearrangments*	*Yes*	*10 / 2*
*MECOM (EVI1)*	*MECOM-rearrangements (but not inv*(3)*)*	*No*	*–*
*MKL1 and RBM15*	*t(1;22) (RBM15-MKL1)*	*Yes*	*1 / 0*
*NOTCH*	*NOTCH-rearrangements (but not t(7;9))*	*No*	*–*
*NUP214*	*t(6;9) (DEK-NUP214)*	*Yes*	*1 / 0*
*PDGFRA*	*del(4q) (FIP1L1-PDGFRA), other PDGFRA-rearrangements*	*Yes*	*1 / 0*
*PDGFRB*	*t(1;5) (PDE4DIP-PDGFRB), t(5;12) (ETV6-PDGFRB), other PDGFRB-rearrangments*	*Yes*	*1 / 0*
*PICALM*	*t(10;11) (PICALM-MLLT10)*	*No*	*–*
*RARA*	*t(15;17) (PML-RARA), t(11;17) (PLZF-RARA), other RARA-rearrangments*	*Yes*	*3 / 1*
*RUNX1*	*t(12;21) (ETV6-RUNX1), t(8;21) (RUNX1-RUNX1T1), t(16;21) (RUNX1-CBFA2T3), other RUNX1-rearrangments*	*Yes*	*3 / 2*
*RUNX1T1*	*t(8;21) (RUNX1-RUNX1T1)*	*Yes*	*1 / 0*
*TAL1*	*del(1p32) (STIL-TAL1)*	*Yes*	*1 / 0*
*TCF3*	*t(1;19) (TCF3-PBX1), t(17;19) (TCF3-HLF)*	*Yes*	*1 / 1*

To evaluate anchored multiplex PCR and NGS-based detection of gene fusions in a clinical setting, diagnostic samples from 27 patients were analyzed. The diagnostic samples were mainly from AML patients but also included ALL, myelodysplastic syndromes (MDS) and myeloproliferative neoplasia (MPN) representing the main genetic subgroups recurrent in hematological malignancies. The gene fusions included in these subgroups are often required for a comprehensive characterization of diagnostic samples.

## Methods

### Patients

Bone marrow or blood samples were collected from 27 patients at the Uppsala University Hospital, Uppsala, Sweden. All cases were classified according to the 2008 WHO classification [1] and samples were collected at diagnosis. In addition, a validation cohort consisting of diagnostic bone marrow or blood samples from 12 patients was included. Slides were prepared from the samples for interphase FISH analysis and from cultured cells for karyotyping and metaphase FISH analysis. Total RNA was prepared from all samples at diagnosis. The study was approved by the ethical board at Uppsala University (Dnr: 2013–233).

### Karyotyping and fluorescence in situ hybridization (FISH)

Cells were cultured and slides were prepared for G-banding according to standard procedures. When possible, metaphases from two cultures were karyotyped. Interphase FISH analysis was performed for screening of genomic aberrations depending on diagnosis and age at diagnosis using either an AML FISH probe panel (including probes for inv/t(16) Vysis LSI *CBFB* Break Apart (BA) rearrangement, t(15;17)(q22;q21) Vysis LSI *PML/RARA* Dual Color, Dual Fusion (DF) Translocation Probe kit, t(8;21)(q21;q22) Vysis LSI *AML1/ETO* Dual Color, DF Translocation Probe and 11q23-rearrangements Vysis LSI *MLL* Dual Color, BA Rearrangement probe, Abbott Laboratories, Chicago, Illinois) or the ALL FISH probe panel (including probes for del(9)(p21) Vysis LSI p16/CEP9, t(1;19)(q23;p13) Vysis LSI *TCF3/PBX1* Dual Color, DF Translocation Probe, t(12;21)(p13;q22) Vysis LSI *ETV6/RUNX1* Dual Color, DF Translocation Probe, t(9;22)(q34;q11) Vysis LSI *BCR/ABL* Dual Color, DF Translocation Probe, 11q23-rearrangements Vysis LSI *MLL* Dual Color, BA Rearrangement probe, Abbott Laboratories, Chicago, Illinois). For specific cases, additional probes were used: Poseidon (Kreatech) *MLL/MLLT1* t(11;19) Fusion Probe (Leica Biosystems, Wetzlar, Germany), Vysis 4q12 Tri-Color rearrangement Probe, Vysis LSI *ETV6* (TEL) Dual Color Probe (Abbott Laboratories,, Chicago, Illinois) and (Kreatech) *MLL/MMLT4* t(6;11) DF-probe (Leica Biosystems, Wetzlar, Germany). The analysis was performed using protocols described by the manufacturers. For each sample, at least 200 interphase nuclei were scored for interphase FISH and for metaphase FISH, at least 10 metaphases were analyzed.

### RNA preparation

RNA was prepared from mononuclear cells using Trizol Reagent Ultra Pure (Invitrogen, ThemoFisher Scientific, Waltham, Massachusetts) according to standard protocols.

### Reverse transcriptase (RT)-PCR

RT-PCR was carried out for the fusions outlined in Table 2 and for the *TCF3*-*ZNF384* e10-e3 fusion. cDNA synthesis was performed using 1.5 μg RNA and M-MLV Reverse Transcriptase according to manufacturer’s instructions (Invitrogen, ThemoFisher Scientific, Waltham, Massachusetts). Primer and probe sequences for PCR are given in 5′- > 3′ orientation: *PML*-*RARA* e6-e3 (Forward primer (F): TCTTCCTGCCCAACAGCAA, Reverse primer (R): GGCTTGTAGATGCGGGGTAG, Probe (P): TAGTGCCCAGCCCTCC); *PML*-*RARA* e3-e3 (F: GACCTCAGCTCTTGCATCACC, R: GGCTTGTAGATGCGGGGTAG, P: TAGTGCCCAGCCCTCC); *RBM15*-*MKL1* e1-e4 (primer-probe mix Hs03024505-ft (Invitrogen, ThemoFisher Scientific, Waltham, Massachusetts)); *KMT2A*-*MLLT4* e8-e2 (F: CCCAAGTATCCCTGTAAAACAAAAA, R: TGCAAAGTTTCCAGCAGCTT); *KMT2A*-*ELL* e9-e2 (primer-probe mix Hs03024474-ft (Invitrogen, ThemoFisher Scientific, Waltham, Massachusetts)); *KMT2A*-*AFF1* e8-e4 (F: CCCAAGTATCCCTGTAAAACAAAAA, R: GAAAGGAAACTTGGATGGCTCA, R: CATGGCCGCCTCCTTTGACAG C); *KMT2A*-*MLLT3* e8-e6 (primer-probe mix Hs03296416-ft (Invitrogen, ThemoFisher Scientific, Waltham, Massachusetts)); *KMT2A*-*ARHGEF12* e6-e22 (F:TAAGCCCAAGTTTGGTGGTC, R: GCGCGCCTTCTGTAGTTC); *KMT2A*-*CBL* e7-e16 (F: AAAAGCAGCCTCCACCACC, R: AGTTGATTCTCCGCGGGAAT, P: TGAAGGTTCCCAAGTTCCCGAGA); *BCR*-*ABL1* e13-e2 (F: TCCGCTGACCATCAATAAGGA, R: CACTCAGACCCTGAGGCTCAA, P: CCCTTCAGCGGCCAGTAGCATCTGA); *ETV6*-*RUNX1* e5-e3 (F: CTCTGTCTCCCCGCCTGAA, R: CGGCTCGTGCTGGCAT, P: TCCCAATGGGCATGGCGTGC); *PBX1*-*TCF3* e16-e3 (F: CCAGCCTCATGCACAACCA, R: GGGCTCCTCGGATACTCAAAA, P: CCCTCCCTGACCTGTCTCGGCC); and *TCF3*-*ZNF384* e10-e3 (F: CCATCTGCATCCTCCTTCTC, R: GGGGATAGAAGGCCAGAAGT). For breakpoint validation of the *KMT2A*-*ARHGEF12* e6-e22 fusion the following primers were used; F1: TAAGCCCAAGTTTGGTGGTC, F2: GCAGTGCTGCAAGATGAGAA, F3: CCGCCCAAGTATCCCTGTAA, R1: GCGCGCCTTCTGTAGTTC, R2: CCAGCGTCTGTTCCTTCATT, R3: CCCATCTCCCACACATTTTC. For breakpoint validation of the *TCF3*-*ZNF384* e10-e3 fusion the following primers were used; F1: CCATCTGCATCCTCCTTCTC, F2: TACTCCCCGGATCACTCAAG, R1: GGGGATAGAAGGCCAGAAGT, R2: CAGGGACCACCGTGATATTC and R3: CCTCGTCCAGGTGGTCTTC. PCR-protocols are available upon request. The RT-PCR breakpoint validations of the *KMT2A*-*ARHGEF12* and the *TCF3*-*ZNF384* fusions were analyzed using 2200 TapeStation, D1000 ScreenTape and the TapeStation Analysis Software version A.02.01 SR1 (Agilent, Santa Clara, California).

**Table 2:** Results from targeted RNA sequencing using Archer™ FusionPlex™ Heme Panel version 1

Aberration^**a**^		Diagnosis	Tissue	FISH (% cells)	Additional method	Transcript	Number of unique reads (% of gene target)
Normal karyotype		AML	BM	NA	NA	*–*	–
		MDS	BM	NA	NA	*–*	–
t(8;21)		AML	BM	85%		*RUNX1-RUNX1T1* e6-e2	1414 (99%)
t(15;17)		AML	BM	56%	RT-PCR	*PML-RARA* e6-e3	337 (29%)
		AML	BM	22%	RT-PCR	*PML-RARA* e3-e3	206 (25%)
		AML	BM	89%	RT-PCR	*PML-RARA* e3-e3	110 (68%)
inv(16)		AML	BM	47%		*CBFB-MYH11* e5-e33	108 (62%)
t(1;22)		AML	PB	NA	RT-PCR	*RBM15-MKL1* e1-e4	442 (41%)
t(6;9)		AML	BM	NA	–	*DEK-NUP214* e9-e18	298 (69%)
*KMT2A*-rearrangement	*KMT2A* PTD	AML	BM	NA	SNP-array	*KMT2A e8e2 fusion*	1024 (19%)
	Unbalanced t(6;11)	AML	BM	83% (del(11q))	RT-PCR	*KMT2A-MLLT4* e8-e2	1538 (76%)
	Unbalanced t(6;11)	AML	BM	80% (del(11q))	RT-PCR	*KMT2A-MLLT4* e8-e2	924 (92%)
	t(11;19)	AML	BM	84%	RT-PCR	*KMT2A-ELL* e9-e2	337 (72%)
	ins(10;11)	AML	BM	28% (del(11q))	–	*KMT2A-MLLT10* e6-e15	86 (74%)
	t(4;11)	B-ALL	BM	94%	RT-PCR	*KMT2A-AFF1* e8-e4	785 (79%)
	t(9;11)	B-ALL	PB	88%	RT-PCR	*KMT2A-MLLT3* e8-e6	431 (44%)
	del(11q23)	B-ALL	BM	87%	RT-PCR	*KMT2A-ARHGEF12* e6-e22	1153 (90%)
	t(11;19)	T-ALL	BM	41%^b^	–	*KMT2A-ENL* e8-e2	313 (30%)
	?t(11;22;11)	T-ALL	BM	73%	RT-PCR	*KMT2A-CBL* e7-e16	120 (59%)
t(9;22)		AML (prev PV)	PB	75%	–	*BCR-ABL1* e1-e3	408 (68%)
		B-ALL	BM	27% (atypical)	RT-PCR	*BCR-ABL1* e13-e2	280 (45%)
t(12;21)		B-ALL	BM	86%	RT-PCR	*ETV6-RUNX1* e5-e3	3215 (15%)
		B-ALL	BM	98%	–	*ETV6-RUNX1* e4-e3	5001 (34%)
t(1;19)		B-ALL	BM	56%	RT-PCR	*TCF3-PBX1* e16-e3	6505 (30%)
del(1)(p32p32)		T-ALL	PB	NA	SNP-array	*STIL-TAL1* e1-e3	53 (10%)
del(4)(q12q12)		MPN	BM	48%	–	*FIP1L1-PDGFRA* e13-e12	341 (76%)
t(5;12)		MPN	BM	87%	–	*ETV6-PDGFRB* e7-e10	432 (97%)

^**a**^*Aberration according to results from chromosome analysis, FISH, RT-PCR and/or SNP-array*

^b^*The gene fusion was also detected with a FISH probe specific for KMT2A-ENL fusion*

### Targeted sequencing

Library preparation was performed with the Archer™ FusionPlex™ Heme Panel v1 with Archer™ Universal RNA Fusion Detection v1 for the Illumina Platform according to the protocols described by the manufacturer (ArcherDX, Boulder, Colorado) (Fig. 1). 200 ng RNA was used as input material. Libraries were purified using Agencourt AMPure Beads on a Life Technologies™ DynaMag™ and quantified with the KAPA Biosystem Library Quantification Kit (Illumina, San Diego, California). Libraries were sequenced by combining four samples, at a concentration of 18pM, using the sequencing kit version 2 and the MiSeq instrument (Illumina, San Diego, California). 10% PhiX was used. Given the size of our clinical laboratory, simultaneous runs of four samples would meet the need to routinely perform the analysis once a week. For the validation cohort, Archer™ FusionPlex™ Heme Panel v2 (ArcherDX, Boulder, Colorado) was used and samples were sequenced in batches of six, using the sequencing kit version 3 and the MiSeq instrument (Illumina, San Diego, California). The Heme Panel v2 was used due to the fact that the v1 panel was no longer commercially available, however, the targets examined are included in both versions.

### Data analysis of sequencing results

Sequencing data were analyzed in the Archer™ Analysis 3.1.1 Software (ArcherDX, Boulder, Colorado). For the validation cohort Archer™ Analysis 6.0.3.2 Software (ArcherDX, Boulder, Colorado) was used as the 3.1.1 Software was not compatible with the Heme Panel version 2. The fusion detection algorithm of strong candidate fusions included mapping of reads to a control region followed by mapping to target regions, the remaining reads were mapped to the human genome (hg19 (GRCh37)). Reads spanning two separate genes were considered fusion candidates if at least 23 bp were mapped on either side of the breakpoint. Each fusion candidate read that spanned the same breakpoint between two reads were binned and a final consensus sequence was compared to the human genome to annotate fusion partners. The following criteria were used in order to qualify a candidate fusion as a strong evidence fusion: i) candidate had a minimum coverage of 5 unique reads; ii) candidate was present in Quiver (if found in Quiver this overrode all subsequent criteria and was reported as a strong evidence fusion); iii) percent of breakpoint-spanning reads of gene-specific primer 2 (GSP2, used in gene-specific PCR 2, see Fig. 1) that supported the candidate relative to the total number of RNA reads spanning the breakpoint was at least 10%; and iv) candidate had at least 3 unique start sites (unique start sites refer to a subset of the unique reads and represent the total number of unique fragment lengths extracted from the sample). The candidate was not considered as a strong evidence fusion if it fulfilled any of the following conditions: i) if it was an exon-intron fusion; ii) if there was evidence of mispriming; iii) if the candidate aligned to known paralogs; iV) if the alignment to the human genome was poor; or v) if cross-contamination to a fusion in the same analysis was present. For a more thorough description of the fusion filters we refer to the Archer Analysis user manual. The QC settings used were: minimum unique reads for valid fusion = 5, minimum average unique RNA start sites per GSP2 controls = 10 (GSP2 control refers to gene-specific primers that target genes that are reliably expressed in any tissue type), minimum unique start sites for valid fusions = 3, fusion percent of GSP2 reads = 10, minimum average unique RNA reads per GSP2 = 0. All filters and cutoffs used were standard settings in the Archer Analysis software.

## Results

### Detection of recurrent gene fusions

**Fig. 2:**
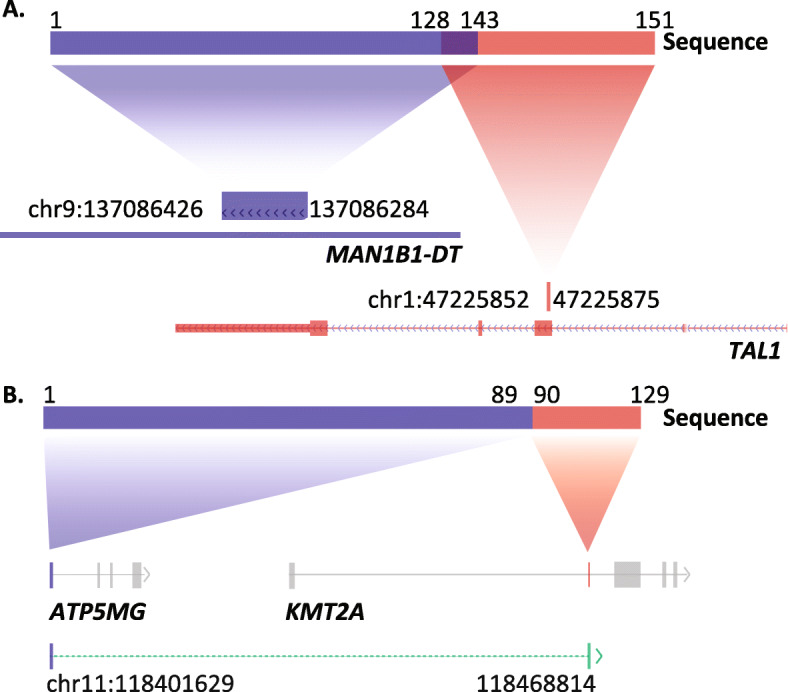
Illustration of artifacts detected by the Archer™ FusionPlex™ Heme Panel version 1 and the Archer™ Analysis Software. **a**. The retrieved sequence read contains sequences that match to a non-coding RNA, *MAN1B1*-*DT*, and the *TAL1* gene. The last part of the sequence contains part of exon 3 of the *TAL1* gene and the noncoding *MAN1B1*-*DT* RNA-transcript with a sequence overlap of 15 bp. **b**. Possible transcription readthrough event between exon 1 of the *ATP5MG* gene and exon 2 of the *KMT2A* gene located downstream of the *ATP5MG* gene

Twenty-seven samples from patients with newly-diagnosed hematological malignancies were selected (14 AML, 7 B-ALL, 3 T-ALL, 2 MPN and 1 MDS) and enriched with Archer anchored multiplex PCR for the Hematology panel and sequenced on a MiSeq instrument (Table 2). To test the clinical utility of the assay, we analyzed cases representing the most recurrent gene fusions of clinical relevance in the panel (Table 1). For most cases, bone marrow was used for the extraction of RNA, except for four cases, where RNA was extracted from peripheral blood. For all cases with known aberrations, as determined by chromosome analysis, FISH analysis, RT-PCR and/or SNParray, the gene fusions could readily be detected by the Archer anchored multiplex PCR and MiSeq sequencing (Table 2). The average number of unique reads among the samples was 1034 (median 408). All except two cases, a T-ALL with a *STIL-TAL1* fusion and an AML with a *KMT2A-MLLT10* fusion, demonstrated more than 100 unique reads spanning the breakpoint of the gene fusion. In short, we could detect the expected fusion genes in all samples carrying recurrent rearrangements. In total, three fusions suspected to be artefacts were reported by the analysis software, all predicted to be out of frame. Two out of three were seen in one case each and demonstrated sequence overlap between the fusion genes. Therefore, they were suspected to be mispriming events or alignment artefacts (*MAN1B1-DT*-*TAL1* and *SRRM2*-*TAL1*) (see Fig. 2a). The third fusion was seen in five cases and contained a fusion between *KMT2A* and a gene 30 kb upstream of *KMT2A*, *ATP5MG*. The fusion was considered a transcriptional readthrough event (see Fig. 2b).

For validation of the primary cohort, samples from 12 patients were analyzed with Archer anchored multiplex PCR and MiSeq. The results were assessed by a clinical molecular geneticist without prior knowledge of diagnosis, karyotype, FISH- or RT-PCR results and scored for fusions. Fusions detected by the FISH panels were all correctly scored by analysis with targeted RNA sequencing, see Table 3. In addition, cases without known fusions according to the FISH panels used were assessed correctly. One case was found to carry a *TCF3*-*ZNF384* fusion using targeted RNA sequencing. The fusion has been reported as a cryptic aberration in ALL [9] and was not detected with the FISH panel used. The fusion and breakpoint of the transcript were verified with RT-PCR.

**Table 3:** Fusion genes detected in the Validation cohort with the Archer™ FusionPlex™ Heme Panel version 2

Aberration according to AML or ALL FISH probe panel	Fusion interpretation of result from targeted RNA sequencing using Anchored multiplex PCR		Concordant with FISH result?
	Transcript	Number of unique reads (% of gene target)	
inv(16); *CBFB-MYH11*	*CBFB-MYH11* e5-e33	2068 (48%)	Yes
t(1;19); *TCF3-PBX1*	*TCF3-PBX1* e16-e3	2216 (50%)	Yes
t(10;11); *KMT2A-MLLT10*	*KMT2A-MLLT10* e9-e8	471 (10%)	Yes
t(15;17); *PML-RARA*	*PML-RARA* e6-e3	1405 (32%)	Yes
t(12;21); *ETV6-RUNX1*	*ETV6-RUNX1* e5-e3	2242 (11%)	Yes
t(12;21); *ETV6-RUNX1*	*ETV6-RUNX1* e5-e3	1656 (18%)	Yes
t(9;22); *BCR-ABL1*	*BCR-ABL1* e1-e2	2084 (49%)	Yes
t(9;11); *KMT2A-MLLT3*	*KMT2A-MLLT3* e8-e6	803 (18%)	Yes
No fusion,signal pattern consistent with iAMP21^a^	No fusion	–	Yes
No fusion	*TCF3*-*ZNF384* e12-e3	564 (54%)	Yes^b^
No fusion	No fusion	–	Yes
No fusion	No fusion		Yes

^a^*iAMP21 confirmed with SNP-array*

^b^*Fusion detected with Archer****™****PCR was not included in the FISH-panel used for analysis of the sample*

**Fig. 3:**
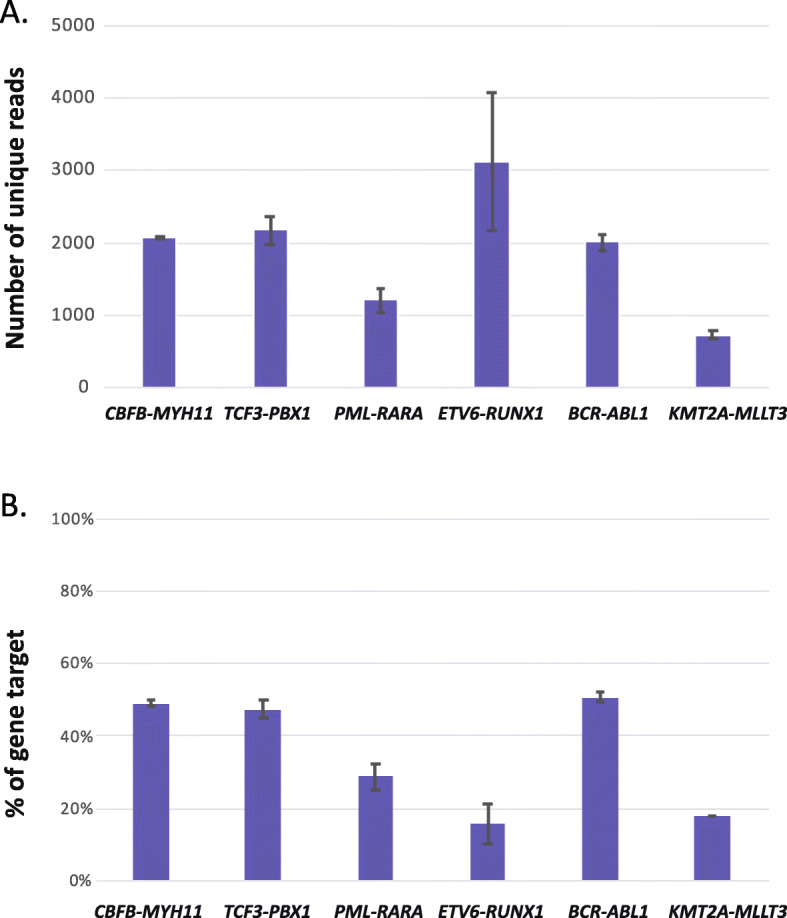
Technical replicates sequenced at three different time points. **a**. Average number of unique reads with standard deviation. **b**. Average percentage of gene target with standard deviation

Technical replicates were performed for six cases with gene fusions (*CBFB*-*MYH11*, *TCF3*-*PBX1*, *PML*-*RARA*, *ETV6*-*RUNX1, BCR*-*ABL1* and *KMT2A*-*MLLT3*). These cases were all sequenced three times at different time points. The gene fusions were detected in all replicates. When comparing the number of unique reads and the percentage of gene targets between technical replicates, a low variation was seen for all fusions except *ETV6*-*RUNX1*, see Fig. 3.

### Identification of rare fusion transcripts

Besides successfully detecting the expected gene fusions, we could identify gene fusions with rare breakpoints that elude detection using routine standard RT-PCR assays. These included two acute leukemia cases, an AML with t(9;22)(*BCR*-*ABL1*) and a B-ALL with t(12;21) (*ETV6*-*RUNX1*) (Table 2). In the t(9;22) case, the routine RT-PCR screening assay included the *BCR-ABL1 major*, *BCR-ABL1 minor* and *BCR-ABL1 micro* fusion transcripts. Anchored multiplex PCR-enriched sequencing identified a gene fusion with an alternative breakpoint, generating a *BCR*-*ABL1* exon 1 and 3 fusion transcript. For the t(12;21) case, the routine RT-PCR assay for the common *ETV6*-*RUNX1* exon 5 and 3 fusion transcript detected amplification. However, the Anchored multiplex PCR-enriched sequencing approach revealed that the patient carried a rare transcript variant *ETV6-RUNX1* exon 4 and 3. Both rare fusion transcripts have been described previously but only in a limited number of cases [10, 11]. In summary, the method could identify rare fusion transcripts otherwise missed by routine RT-PCR screening assays.

### Identification of *KMT2A* fusions

**Fig. 4:**
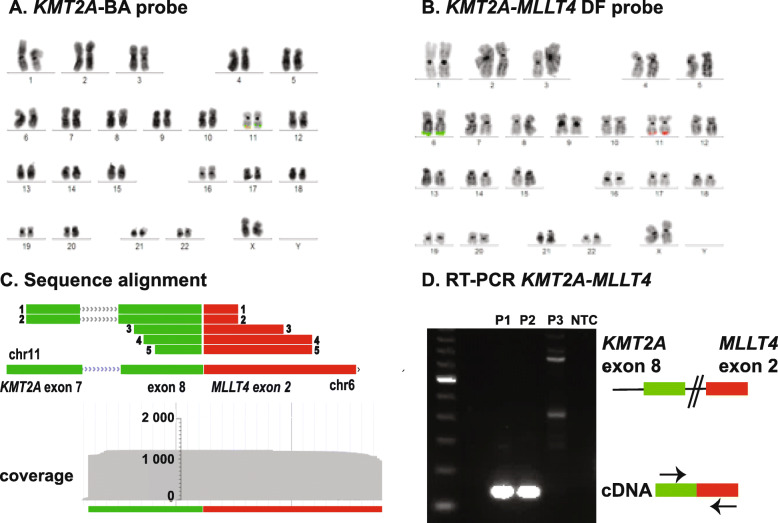
A cryptic *KMT2A*-rearranged AML. The figure shows an AML with a *KMT2A*-*MLLT4* gene fusion which is likely caused by an unbalanced translocation between chromosome 6 and 11. **a**. FISH-analysis using the *KMT2A* BA-probe (*KMT2A* 5′ = green FISH-probe, *KMT2A* 3′ = red FISH-probe) could detect that a suspected *KMT2A*-rearrangement was present since deletion of the 3′-part (red) of the *KMT2A*-gene was seen. However, because of the lack of the reciprocal fusion, no fusion partner could be identified. **b**. The translocation was not visible with G-banding or FISH-analysis using *KMT2A*/*MLLT4* dual fusion-probe (*KMT2A* = red FISH-probe, *MLLT4* = green FISH-probe). **c**. Archer anchored multiplex PCR and MiSeq sequencing revealed a *KMT2A*-*MLLT4* exon 8-exon 2 fusion. The figure is a schematic overview of the sequences, a total of 924 reads spanning the breakpoint was scored. **d**. RT-PCR verified the *KMT2A*-*MLLT4* gene fusion. P1 and P2 = patient 1 and 2 carrying *KMT2A*-*MLLT4* e8-e2 gene fusions, P3 = patient 3 with a *KMT2A*-*AFF1* gene fusion (negative control), NTC = non template control. For the original full length gel image see Supplementary Fig. 1

Due to their complexity, we chose to analyze eight cases with *KMT2A*-rearrangements, representing seven different fusion partners (Table 2). Furthermore, we included a case with a *KMT2A* partial tandem duplication (PTD). Notably, all gene fusions in all cases could be readily identified using the Anchored multiplex PCR-enriched sequencing approach. Importantly, four cases were found to have cryptic *KMT2A*-rearrangements where the fusion partner could not be determined with conventional methods (for examples, see Figs. 4 (Supplementary figure 1) and 5). Of the four cryptic *KMT2A*-rearrangements, two of these were *KMT2A-MLLT4* fusions, which were most likely the result of unbalanced translocations between the long arms of chromosome 6 and 11. In these cases, only the fusion at chromosome 11 was present, whereas the reciprocal fusion on chromosome 6 was missing. These fusions could not be detected with gene-specific FISH, but RT-PCR could readily verify the rearrangements detected by NGS-sequencing. The third case was an interstitial deletion on the long arm of chromosome 11, causing the *KMT2A*-gene to fuse with the *ARHGEF12-*gene distal to the *KMT2A*-gene on chromosome 11. Of note, this fusion event would not be identified with conventional methods and is likely under-diagnosed in acute leukemia. The *KMT2A*-*ARHGEF12* fusion was verified with RT-PCR. To further investigate the breakpoint of this rare fusion, several primers sets were used in RT-PCR, generating various expected fragment sizes. The results verified the transcript breakpoint reported from analysis of the Anchored multiplex PCR-enriched sequencing (Fig. 6 (Supplementary figure 2)).

**Fig. 5:**
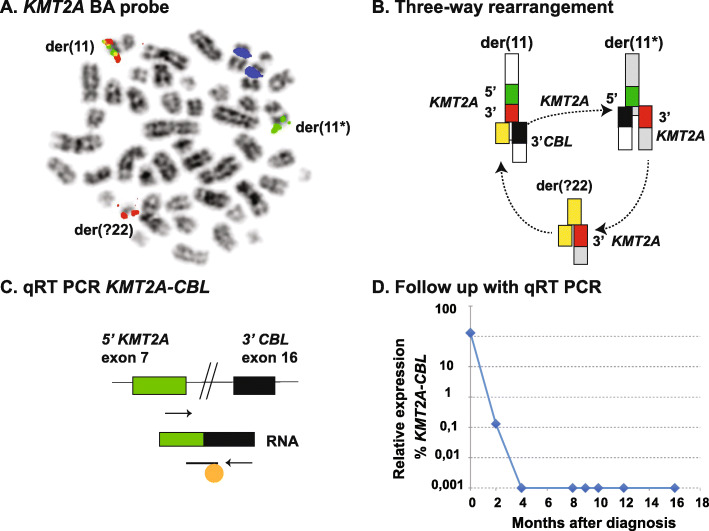
Development of a MRD follow up assay for T-ALL patient with a *KMT2A*-*CBL* fusion. **a**. The rearrangement was not detectable with G-banding but was with FISH using the *KMT2A* BA-probe (*KMT2A* 5′ = green FISH-probe, *KMT2A* 3′ = red FISH-probe). The 3′ part of *KMT2A* (red) was found to be translocated to another chromosome. **b**. Archer anchored multiplex PCR revealed a *KMT2A*-*CBL* fusion (likely a result of a three-way translocation as the distal part of *KMT2A* had translocated to another chromosome). In the figure the genes and chromosomes are illustrated as follows: *KMT2A* 5′ = green; *KMT2A* 3′ = red; *CBL* = black; unidentified derived chromosome (der(?22)) = yellow. **c**. and **d**. The transcript information from the targeted RNA sequencing could be used for design of primers and probes for qPCR. Arrows = forward and reverse primers. Line with orange ball = fluorescently-labelled TaqMan probe

**Fig. 6:**
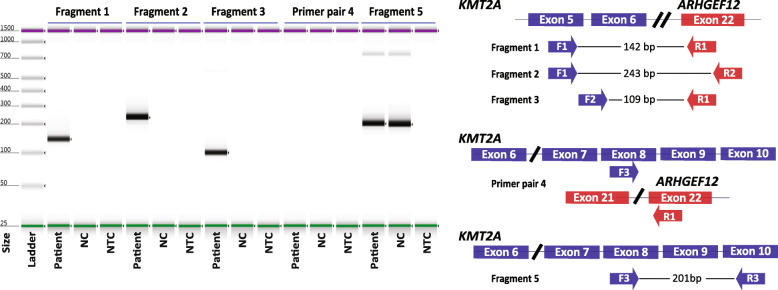
Verification of the *KMT2A* exon 6-*ARHGEF12* exon 22 fusion breakpoint. RT-PCR results (ScreenTape) and schematic overview of primer location with expected fragment size according to the breakpoint defined by RNA-sequencing with the ArcherTM FusionPlex™ Heme Panel. Sample is from a patient with a *KMT2A*-*ARHGEF12* fusion. NTC = non template control. Arrows with F1-F3: forward primers. Arrows with R1-R3: reverse primers. For the original full length ScreenTape image see Supplementary Fig. 2

### Development of a minimal residual disease follow-up assay for a patient with *KMT2A* fusion using the fusion transcript sequence

In the fourth case with a cryptic *KMT2A* fusion, no visible chromosomal aberrations were detected by G-banding, however, FISH analysis showed a *KMT2A* break apart pattern. Metaphase FISH showed the distal part of the *KMT2A*-gene on a chromosome in the G-group, likely chromosome 22 (Fig. 5). Anchored multiplex PCR enriched sequencing demonstrated a fusion between *KMT2A* and *CBL*, a gene downstream of *KMT2A* on chromosome 11. Using the fusion transcript sequence acquired in the RNA sequencing, a primer-probe assay specific for the patient could be designed and used for minimal residual disease (MRD) detection of the patient (Fig. 5). In summary, the method can identify *KMT2A* fusion partners in cryptic rearrangements and can provide sequence information which enables the design of patient-specific follow-up RT-PCR assays.

## Discussion

The clinical laboratory constantly strives to gain a deeper genetic characterization of patients at increased efficiency and lower cost. The ever-decreasing cost of NGS-based technologies is currently paving the way for the widespread adoption of such platforms in the clinical space [12]. As new technologies emerge and evolve, strict validation of such platforms is imperative for implementation in the clinical diagnostic setting. Validations of targeted RNA sequencing of gene fusion panels in Childhood sarcoma (ChildSeq) and CNS tumors (GlioSeq) have been published [13, 14]. Also, the Anchored multiplex PCR solid cancer gene fusion panel, the Pan-Heme panel and the TruSight RNA fusion panel have been validated [15–17]. Qu et al performed a comparison of four NGS platforms for fusion detection: Oncomine, AmpliSeq, QIAseq and Anchored multiplex PCR solid cancer gene fusion panel [18]. In a recent study the Anchored multiplex PCR heme panel version 2 was investigated for detection of ten different *KMT2A*-rearrangements [19]. Here, we show that targeted RNA sequencing can also be used to screen for other recurrent gene fusions in acute leukemia and related hematological malignancies on diagnostic samples using a time-saving protocol.

According to the WHO Classification of AML, the diagnosis of a *KMT2A*-rearranged leukemia should specify the fusion partner [1]. One third of *KMT2A* translocations cannot be detected by conventional karyotyping and require FISH or RT-PCR [20]. Thus, identification of the fusion partner of the *KMT2A*-gene in routine diagnostics often requires metaphase FISH, FISH with fusion-specific probes or RT-PCR with transcript-specific primers. This type of screening is time-consuming and fails to identify the less common *KMT2A*-fusions. In agreement with Afrin et al, we have demonstrated that targeted RNA-sequencing by anchored PCR can function as a true screening method, identifying any gene connected to the *KMT2A* gene without any prior knowledge of the transcript [19]. We could successfully demonstrate this for a case which showed a 20 Mb deletion on the long arm of chromosome 11, joining the *KMT2A*-gene with the *ARHGEF12*-gene (Table 2). To our knowledge, only two cases have been reported with this gene fusion [20, 21]. The *KMT2A-ARHGEF12* fusion is most likely more common but is missed due to the limitations of chromosome analysis, FISH and RT-PCR approaches. The function of the chimeric proteins in *KMT2A*-rearranged leukemia is not entirely understood, but *KMT2A* fusion proteins have been shown to interfere with transcriptional elongation and thereby deregulate expression of target genes [5]. Several studies have demonstrated the potential use of *KMT2A* inhibitors as promising targeted therapies for *KMT2A*-rearranged leukemia [22, 23]. Thus, correctly identifying and characterizing *KMT2A-*rearrangements is of the utmost importance for 1) leukemia risk stratification and 2) choice of therapy.

Targeted RNA sequencing enabled us to detect rare transcript variants of the commonly-occurring gene fusions *BCR-ABL1* and *ETV6-RUNX1,* which might otherwise be missed by RT-PCR approaches. Similarly, less common gene fusions, or genes with several fusion partners were identified. Using amplicon-based transcript enrichment strategies, these rare transcript variants or gene fusions would not have been detected, highlighting the limitations of such strategies and the need to transition away from their use as stand-alone approaches in the screening of clinical samples.

As expected, large variations in read depth were seen for the different gene fusions. This was likely due to variation in the number of cells carrying the gene fusion in the diagnostic samples, differences in expression levels of the gene fusion and the efficiency of the anchored PCRs. In addition, the expressed wild type genes also compete with the number of reads. Technical sequencing replicates of six cases showed low variation in the number of unique reads for all fusions tested, except *ETV6*-*RUNX1* (Fig. 3). *ETV6*-*RUNX1* were highly expressed with a higher number of reads compared to the other targets. This may contribute to a larger variation between sequencing runs. Overall, we detected many more reads per fusion when compared to published data where non-targeted RNA sequencing has been used to detect gene fusions. A study applying RNA sequencing on 179 AML patients detected, on average, 40 reads per total detected fusion and 49 reads per in-frame fusion [7]. Similarly, using RNA sequencing, Liljebjörn et al identified clinically relevant fusion genes in leukemic cell lines, but in the majority of samples only a few reads representing gene fusions were found [24]. In 6 out of 15 cell lines, fewer than 10 reads were scored per fusion. In addition, the bioinformatic analysis required SNP array data to filter for fusions and as much as 26% of the fusions could not be verified as genuine gene fusions with RT-PCR or Sanger sequencing. Furthermore, it is difficult to estimate the number of false positives that arise using RNA sequencing as all fusions recovered at similar levels as true fusions have not been systematically assessed by RT-PCR. Panagopoulos et al highlighted the risk of missing pathogenic essential gene fusions in patients when using transcriptome sequencing combined with bioinformatics algorithms as a stand-alone technique [25]. In a clinical diagnostic setting, a low number of reads would require verification of the gene fusion with an additional method such as RT-PCR or FISH. However, one drawback of the targeted sequencing approach is that novel fusions of genes not included in the panels will be missed. The knowledge of somatic genetic aberrations of leukemia patients is rapidly increasing as more NGS data are collected. In an RNA sequencing study of 195 pediatric B-ALL cases, 65% had in-frame gene fusions, of which 27 were novel fusions [26]. This highlights the need for efficient and robust laboratory methods for detection of genetic aberrations in clinical practice, including gene fusions, without prior knowledge of the patients karyotype or genome. As the discovery of novel gene fusions saturates, it will be possible to design comprehensive targeted gene panels that fulfill the requirements of a clinical routine diagnostic laboratory. Ideally, a panel should include relevant spike-in controls to accurately monitor sensitivity and specificity in each sequencing run.

One drawback of the method used in this study is the use of nested PCR, which makes the assay sensitive to residual PCR products that can be amplified in the second PCR. This requires the use of separate rooms during the library preparation process and of UV-light or chemical destruction for elimination of contaminating PCR products. In light of this, the approach should mainly be used at diagnosis and not as an MRD method. Nevertheless, as the sequencing provides transcript-specific information for each gene fusion design of MRD assays for careful follow up of patients is feasible, e.g. qPCR, a method with a reported sensitivity of 10^− 5^ [27]. In this study, we demonstrate how this can be achieved.

## Conclusion

To summarize, we have shown that targeted RNA sequencing using Archer anchored multiplex PCR can be applied for the detection of recurrent gene fusions in hematological malignancies in a clinical setting. All fusions known to be present in previously tested patient samples could successfully be identified with the method. In addition, cases analyzed without prior knowledge of karyotype or diagnosis were correctly assessed. The use of targeted RNA sequencing simplifies gene fusion screening, can easily be implemented to complement FISH-analysis routinely used in leukemia diagnostics and facilitates identification and design of patient-specific MRD assays. Furthermore, targeted RNA sequencing can be used to investigate patients where only small amounts of diagnostic material are available.

## Supplementary information

**Additional file 1 Figure S1** Original image of the agarose gel in Fig. 4d showing the RT-PCR result of the *KMT2A*-*MLLT4* gene fusion. P1 and P2 = patient 1 and 2 carrying *KMT2A*-*MLLT4* e8-e2 gene fusions, P3 = patient 3 with a *KMT2A*-*AFF1* gene fusion (negative control), NTC = non template control. **Figure S2** Original image of the ScreenTape result and expected fragment sizes from the TapeStation analysis of the breakpoint verification of the *KMT2A* exon 6-*ARHGEF12* exon 22 fusion breakpoint using RT-PCR from Fig. 6. Sample is from a patient with a *KMT2A*-*ARHGEF12* fusion. NC = negative control (cDNA from patient with no *KMT2A*-*ARHGEF12* fusion). NTC = non template control. Arrows with F1-F3: forward primers. Arrows with R1-R3: reverse primers.

## Data Availability

The RNA sequencing data generated during the current study are available in the NCBI Read Archive and searchable in SRA Run Selector, BioProject ID PRJNA637231. All results are presented relative to hg19/GRCh37 (Genome Reference Consortium Human Reference 37, GenBank assembly accession: GCA_000001405.1).

## Abbrevations

ALL: Acute lymphoblastic leukemia
AML: Acute myeloid leukemia
CML: Chronic myeloid leukemia
FISH: Fluorescence in situ hybridization
MDS: Myelodysplastic syndromes
MPN: Myeloproliferative neoplasia
MRD: Minimal residual disease
PTD: Partial tandem duplication
RT: Reverse transcriptase

## References

[1] Swerdlow SH, Campo E, Harris NL, Jaffe ES, Pileri SA, Stein H, Thiele J, Vardiman JW (2008). WHO Classification of Tumours of Haematopoietic and Lymphoid Tissues.

[2] Rowley JD (1973). A new consistent chromosomal abnormality in chronic Myelogenous Leukaemia identified by Quinacrine fluorescence and Giemsa staining. Nature..

[3] Baccarani M, Deininger MW, Rosti G, Hochhaus A, Soverini S, Apperley JF (2013). European LeukemiaNet recommendations for the management of chronic myeloid leukemia: 2013. Blood..

[4] Yoshida H, Kitamura K, Tanaka K, Omura S, Miyazaki T, Hachiya T (1996). Accelerated degradation of PML-retinoic acid receptor α (PML-RARA) Oncoprotein by all-trans-retinoic acid in acute Promyelocytic leukemia: possible role of the proteasome pathway. Cancer Res.

[5] Tamai H, Inokuchi K (2010). 11q23/MLL acute leukemia : update of clinical aspects. J Clin Exp Hematopathology.

[6] Meyer C, Burmeister T, Gröger D, Tsaur G, Fechina L, Renneville A (2018). The MLL recombinome of acute leukemias in 2017. Leukemia..

[7] The Cancer Genome Atlas Research Network (2013). Genomic and Epigenomic landscapes of adult De novo acute myeloid leukemia. N Engl J Med.

[8] Andersson AK, Ma J, Wang J, Chen X, Gedman AL, Dang J (2015). The landscape of somatic mutations in infant MLL-rearranged acute lymphoblastic leukemias. Nat Genet.

[9] Hirabayashi S, Ohki K, Nakabayashi K, Ichikawa H, Momozawa Y, Okamura K (2017). ZNF384-related fusion genes define a subgroup of childhood B-cell precursor acute lymphoblastic leukemia with a characteristic immunotype. Haematologica..

[10] López-Andrade B, Sartori F, Gutiérrez A, García L, Cunill V, Durán MA (2015). Acute lymphoblastic leukemia with e1a3 BCR/ABL fusion protein. A report of two cases. Exp Hematol Oncol.

[11] Zaliova M, Meyer C, Cario G, Vaskova M, Marschalek R, Stary J (2011). TEL/AML1-positive patients lacking TEL exon 5 resemble canonical TEL/AML1 cases. Pediatr Blood Cancer.

[12] Matthijs G, Souche E, Alders M, Corveleyn A, Eck S, Feenstra I (2016). Guidelines for diagnostic next-generation sequencing. Eur J Hum Genet.

[13] Nikiforova MN, Wald AI, Melan MA, Roy S, Zhong S, Hamilton RL (2016). Targeted next-generation sequencing panel (GlioSeq) provides comprehensive genetic profiling of central nervous system tumors. Neuro-Oncology..

[14] Qadir MA, Zhan SH, Kwok B, Bruestle J, Drees B, Popescu O-E (2014). ChildSeq-RNA: a next-generation sequencing-based diagnostic assay to identify known fusion transcripts in childhood sarcomas. J Mol Diagnostics..

[15] Helm S, Ras A, Spotlow V, Kelly K, Mockus S, Statz C (2016). Abstract 3630: validation of the archer FusionPlex solid tumor panel in the JAX cancer treatment profile. Cancer Res.

[16] Kim B, Lee H, Shin S, Lee S-T, Choi JR (2019). Clinical evaluation of massively parallel RNA sequencing for detecting recurrent gene fusions in hematologic malignancies. J Mol Diagnostics.

[17] Stengel A, Nadarajah N, Haferlach T, Dicker F, Kern W, Meggendorfer M (2018). Detection of recurrent and of novel fusion transcript in myeloid malignancies by targeted RNA sequencing. Leukemia..

[18] Qu X, Yeung C, Coleman I, Nelson PS, Fang M (2020). Comparison of four next generation sequencing platforms for fusion detection: Oncomine by ThermoFisher, AmpliSeq by Illumina, FusionPlex by ArcherDX, and QIAseq by QIAGEN. Cancer Genet.

[19] Afrin S, Zhang CRC, Meyer C, Stinson CL, Pham T, Bruxner TJC (2018). Targeted next-generation sequencing for detecting MLL gene fusions in leukemia. Mol Cancer Res.

[20] Ly S, Liang D, Fu Jf WJ, Wang P, Lin T (2005). Characterization of fusion partner genes in 114 patients with de novo acute myeloid leukemia and MLL rearrangement. Leukemia..

[21] Kourlas PJ, Strout MP, Becknell B, Veronese ML, Croce CM, Theil KS (2000). Identification of a gene at 11q23 encoding a guanine nucleotide exchange factor: evidence for its fusion with MLL in acute myeloid leukemia. Proc Natl Acad Sci U S A.

[22] Daigle SR, Olhava EJ, Therkelsen CA, Majer CR, Sneeringer CJ, Song J (2011). Selective killing of mixed lineage leukemia cells by a potent small-molecule DOT1L inhibitor. Cancer Cell.

[23] Grembecka J, He S, Shi A, Purohit T, Muntean AG, Sorenson RJ (2012). Menin-MLL inhibitors reverse oncogenic activity of MLL fusion proteins in leukemia. Nat Chem Biol.

[24] Lilljebjorn H, Agerstam H, Orsmark-Pietras C, Rissler M, Ehrencrona H, Nilsson L (2014). RNA-seq identifies clinically relevant fusion genes in leukemia including a novel MEF2D/CSF1R fusion responsive to imatinib. Leukemia..

[25] Panagopoulos I, Torkildsen S, Gorunova L, Tierens A, Tjønnfjord GE, Heim S (2014). Comparison between karyotyping-FISH-reverse transcription PCR and RNA- sequencing-fusion gene identification programs in the detection of KAT6A-CREBBP in acute myeloid leukemia. PLoS One.

[26] Lilljebjörn H, Henningsson R, Hyrenius-Wittsten A, Olsson L, Orsmark-Pietras C, von Palffy S (2016). Identification of ETV6-RUNX1-like and DUX4-rearranged subtypes in paediatric B-cell precursor acute lymphoblastic leukaemia. Nat Commun.

[27] Hokland P, Ommen HB, Nyvold CG, Roug AS (2012). Sensitivity of minimal residual disease in acute myeloid leukaemia in first remission – methodologies in relation to their clinical situation. Br J Haematol.

