# Supplementary material for: Detection of leukemia gene fusions by targeted RNA-sequencing in routine diagnostics
Source: BMC Med Genomics. 2020 Jul 29;13:106. doi: 10.1186/s12920-020-00739-4 (PMC7388219; doi:10.1186/s12920-020-00739-4)
Supplement: Supplementary file 1 — Additional file 1 Figure S1 Original image of the agarose gel in Fig. 4d showing the RT-PCR result of the KMT2A-MLLT4 gene fusion. P1 and P2 = patient 1 and 2 carrying KMT2A-MLLT4 e8-e2 gene fusions, P3 = patient 3 with a KMT2A-AFF1 gene fusion (negative control), NTC = non template control. Figure S2 Original image of the ScreenTape result and expected fragment sizes from the TapeStation analysis of the breakpoint verification of the KMT2A exon 6-ARHGEF12 exon 22 fusion breakpoint using RT-PCR from Fig. 6. Sample is from a patient with a KMT2A-ARHGEF12 fusion. NC = negative control (cDNA from patient with no KMT2A-ARHGEF12 fusion). NTC = non template control. Arrows with F1-F3: forward primers. Arrows with R1-R3: reverse primers. [file 12920_2020_739_MOESM1_ESM.docx]

**Supplementary figures**


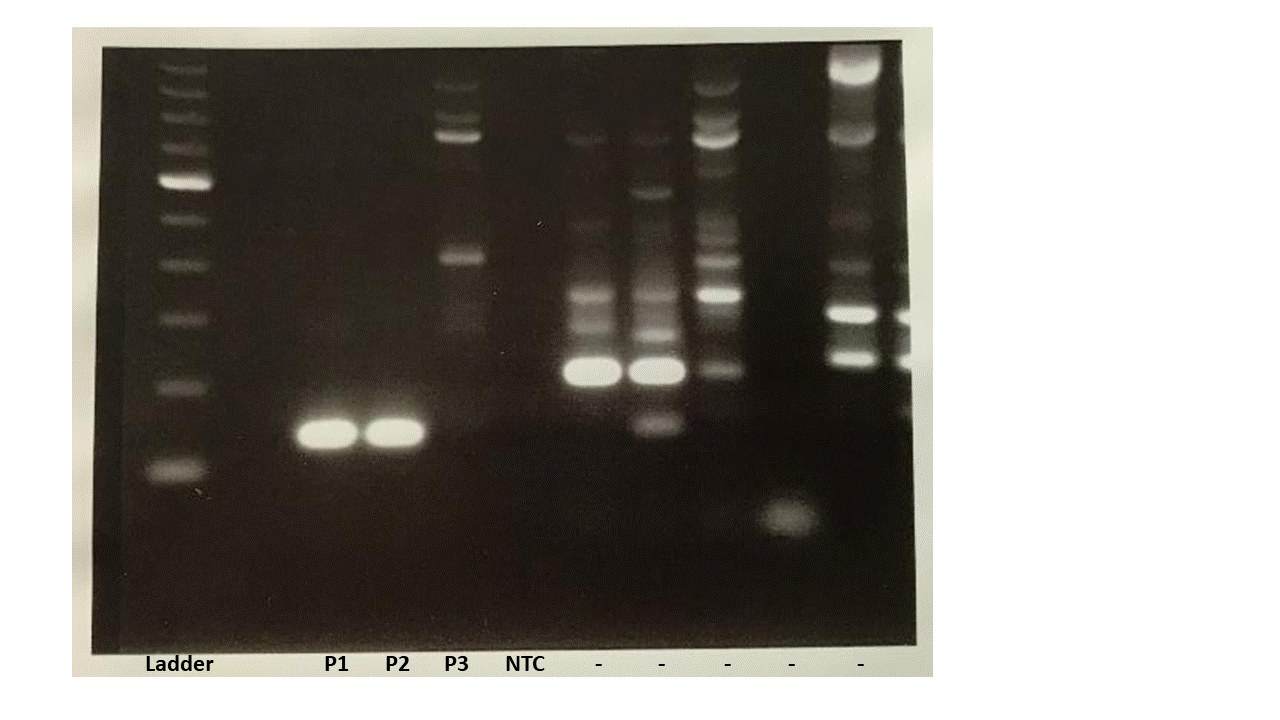


**Supplementary Figure 1.** Original image of the agarose gel in Figure 4D showing the RT-PCR result of the *KMT2A*-*MLLT4* gene fusion. P1 and P2 = patient 1 and 2 carrying *KMT2A*-*MLLT4* e8-e2 gene fusions, P3 = patient 3 with a *KMT2A*-*AFF1* gene fusion (negative control), NTC = non template control.


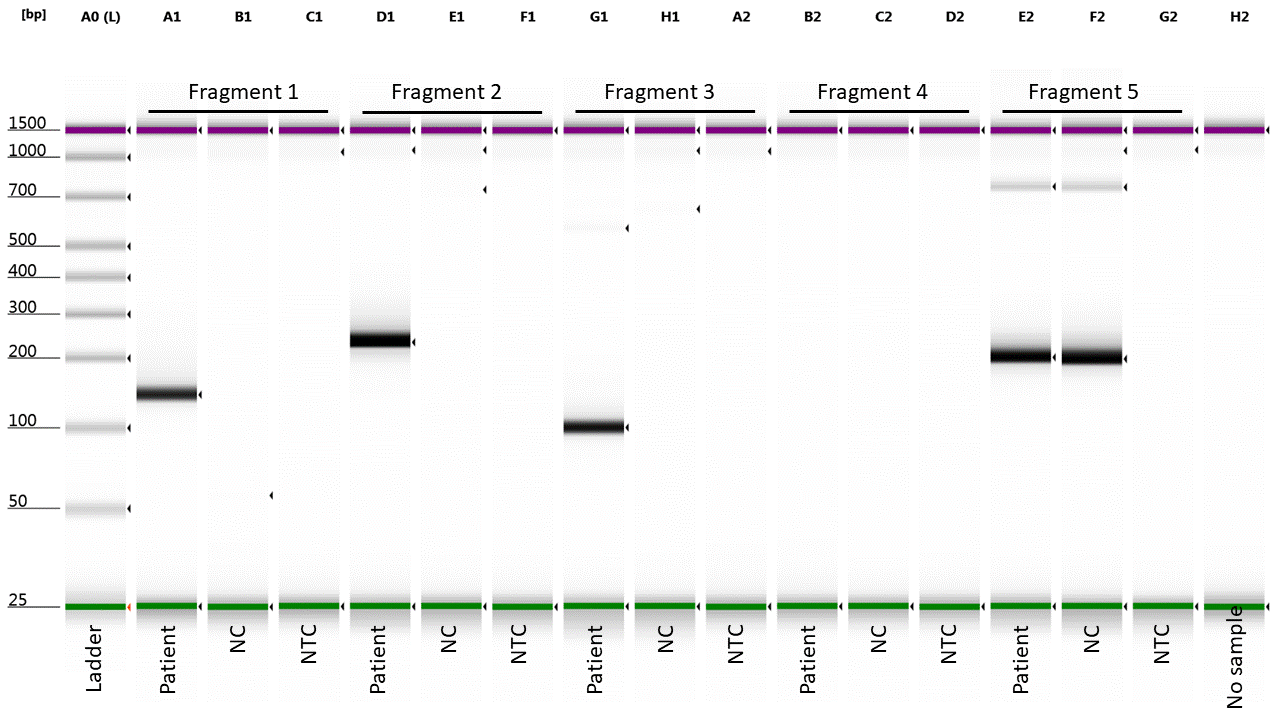

**Supplementary Figure 2.** Original image of the ScreenTape result and expected fragment sizes from the TapeStation analysis of the breakpoint verification of the *KMT2A* exon 6-*ARHGEF12* exon 22 fusion breakpoint using RT-PCR from Figure 6. Sample is from a patient with a *KMT2A*-*ARHGEF12* fusion. NC = negative control (cDNA from patient with no *KMT2A*-*ARHGEF12* fusion). NTC = non template control. Arrows with F1-F3: forward primers. Arrows with R1-R3: reverse primers.
